# Guidelines for the diagnosis and management of food allergy in the United States

**DOI:** 10.1186/2045-7022-1-S1-S10

**Published:** 2011-08-12

**Authors:** Matthew Fenton

**Affiliations:** 1 National Institutes of Health USA, Bethesda, USA

## 

Food allergy is an important public health problem that affects approximately 5 percent of children and 4 percent of adults and may be increasing in prevalence. Despite the risk of severe allergic reactions and even death, there is no current treatment for food allergy: the disease can only be managed by allergen avoidance or treatment of symptoms. The diagnosis and management of food allergy may vary from one clinical practice setting to another, and patients frequently confuse non-allergic food reactions, such as food intolerance, with food allergies. In response to these concerns, the National Institute of Allergy and Infectious Diseases (NIAID, part of the US National Institutes of Health), working with 34 professional organizations, federal agencies, and patient advocacy groups, led the development of clinical guidelines for the diagnosis and management of food allergy. These guidelines are intended for use by a wide variety of healthcare professionals. Topics addressed include the epidemiology, natural history, diagnosis, and management of food allergy, as well as the management of severe symptoms and anaphylaxis. The guidelines were published in December 2010, but Dr. Matthew J. Fenton, from the NIAID, will provide a summary of the guidelines and the methods used to draft them.

